# Dupuytren’s disease: using needles more across the world

**DOI:** 10.1177/17531934211043307

**Published:** 2021-09-09

**Authors:** David Warwick, Paul NM Werker, Gary Pess, Hitoshi Hirata, David J Hunter-Smith

**Affiliations:** 1 University Hospital Southampton, Southampton, UK; 2 Department of Plastic Surgery, University of Groningen and University Medical Centre Groningen, Groningen, The Netherlands; 3 Central Jersey Hand Surgery, Eatontown, NJ, USA; 4 Department of Hand Surgery, Graduate School & Faculty of Medicine Nagoya University, Nagoya, Japan; 5 Department of Plastic, Reconstructive and Hand Surgery, Peninsula Clinical School at Monash University, Melbourne, Australia

**Keywords:** Dupuytren’s disease, percutaneous needle fasciotomy, surgical release, decision-making

## Abstract

In this article we take an international perspective on the use of needles, either percutaneous needle fasciotomy (PNF) or Clostridial Collagenase Histiolyticum (CCH), in treating Dupuytren’s Disease (DD). Worldwide, PNF is now used more frequently. The CCH has been withdrawn from non-USA markets, which lessens its use. Different patients have different preferences, while different surgeons have different skills and opinions. The surgeon should fully consider the patient’s preference and should also, in view of the scarcity of surgical resource and the potential hazard of surgery, reconsider and expand the use of a needle rather than an operation. In the future, a cheaper, yet equally safe and effective alternative to CCH, will provide a useful clinical tool for those cords, which, in the surgeon’s personal Venn diagram, are too challenging for PNF, but the patient does not want to have surgery.

## Introduction

We review the use of needles – either percutaneous needle fasciotomy (PNF) or Clostridial Collagenase Histiolyticum (CCH) – in treating Dupuytren’s Disease (DD). This topic is relevant for two reasons. First, the withdrawal of CCH from the world markets (apart from the USA) has removed a useful treatment tool that avoided surgery for many patients. Second, the pandemic in 2020 has led to a surge in people waiting for treatment for DD: an increased use of needles would free precious operating theatre facilities for other more clinically pressing needs.

**Figure 1.**
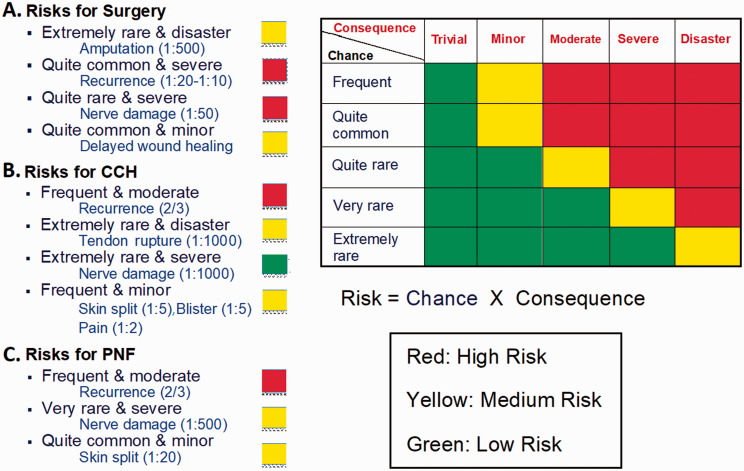
Schematic risk profile of PNF (a), CCH (b) and surgery (c).

Needles are generally safer and have different profiles of risk compared with surgery – either limited fasciectomy (LF) or dermofasciectomy with skin grafting (DF) (Crean et al., 2011; Denkler et al., 2017; Herrara et al., 2015; Leung et al., 2019; Nydick et al., 2013; Peimer et al., 2014; Pess et al., 2012;. Van Rijssen et al., 2006) (Figure 1). There is also a mutually exclusive balance, since needles are intrinsically safer than surgery with a quicker recovery, yet much higher recurrence because no tissue is removed.

## Percutaneous needle fasciotomy

**Figure 2.**
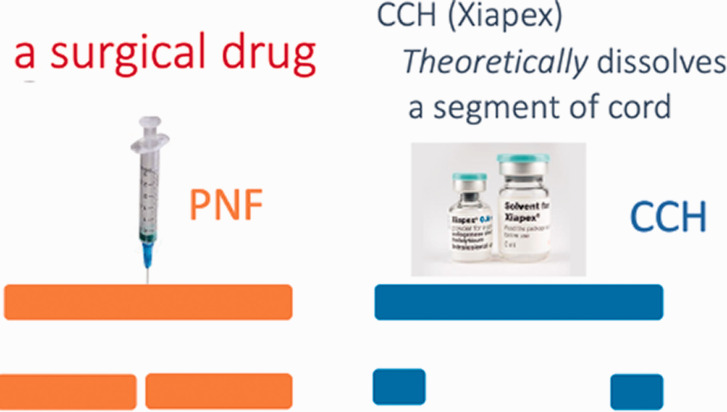
Mode of action of PNF versus CCH.

The modern era of minimally invasive treatments for Dupuytren Contracture began in 1972 with the first publication on needle aponeurotomy (needle aponeurotomy (NA), needle fasciotomy (NF), percutaneous needle fasciotomy (PNF)) by Lermusiaux and Debeyre (1979). They described using a needle to divide the cords. NA was less invasive than open fasciectomy, (Badois et al., 1993a,b; Foucher et al., 2003) subsequently reported on the use of fasciotomy and its comparison to LF, showing a quicker recovery of function and its acceptance increased (Figure 2). This was subsequently supported with the work of Eaton (2011) and Pess et al. (2012).

Since cords are insensate, only very small aliquots of intradermal local anaesthesia are needed at the site of skin puncture to be able to perform the procedure. Cords should always be divided where they are the thinnest and not attached to the skin. With experience, the circle for PNF on the Venn diagram enlarges and the circle for LF shrinks, because PNF may also be performed on thicker cords and at multiple levels from the tip of the finger to the palm during the same session. By doing this and working from distal to proximal, nerve conduction is maintained, enabling the patient to warn the surgeon if the needle touches a nerve.

PNF is as effective in correcting the contracture as the more invasive surgical procedures for cords that affect the metacarpo-phalangeal (MCP) joint, but less so for proximal interphalangeal (PIP) joint contractures (van Rijssen et al., 2006). The efficacy of a first PNF is comparable with that of LF when the initial total passive extension deficit (TPED) of a ray is less than 90° (van Rijssen et al., 2006). Typically, patients can resume normal use of their hand a day after surgery and are advised to refrain from heavy labour for 2 weeks. The cumulative risk of serious local complication of PNF is much less than that of the more invasive treatments, and the risk of damaging nerves or tendons is far less than 0.1%. Skin fissures are more likely to occur, especially with adherent skin, and one can try to prevent this by releasing the skin from the cord before dividing it using a thicker hypodermic needle. Small skin tears usually heal quickly, and do not bother the patient. Bigger tears may need a transposition flap during the same session.

PNF recurrence rates are relatively high compared with those of the open surgery (85% for PNF compared with 21% after LF), but not every recurrence needs re-treatment (van Rijssen et al., 2012). Alser et al. (2020) found that the overall 10-year probability of having reoperation was 33.7% (95% CI 31.4–36.2) after primary PNF, 19.5% (95% CI 18.9–20.1) after primary LF and 18.2% (95% CI 15.7–21.1) after primary DF.

PNF may be safely and successfully used for the treatment of recurrences (van Rijssen and Werker, 2012). PNF can also be used to determine the aggressiveness of the disease: the most benign form can often be retreated with PNF repeatedly, while the less benign form will recur earlier and may not respond as favourably to a second PNF and may benefit from a more aggressive approach. In general, at least PNF postpones more aggressive treatment to a higher age, where recurrence may occur after a longer time frame (van Rijssen et al., 2012).

## Clostridial collagenase histiolyticum

CCH (Xiaflex, Xiapex) is an enzymatic treatment studied extensively by Hurst and Badalamente7-14 and has now been used over 150,000 fingers around the world The drug is a mixture of AUX 1 and AUX 2 collagenase that work synergistically to provide hydrolysing activity and rapidly degrade the collagen in DD. CCH was approved for use in Europe and the United States in 2010. A Supplemental Biologics License Application was approved on 20 October 2014, allowing the treatment of up to two joints in a single treatment visit and permitting delayed manipulation 24–72 hours after injection.

The technique for administering CCH is taught in two-thirds of American Hand Fellowships and the use continues to increase in the USA (Personal communication, Endo Pharmaceuticals, 2019). The increase in demand is a combination of patient demand for a non-surgical alternative and the increase in a physician’s comfort level and skill with repeated injections.

However, in 2019, Endo Pharmaceuticals withdrew the marketing licence for all-world markets apart from the USA, because the demand for the product remained unsustainably low in non-USA markets.

When PNF was introduced in the USA in 2004, most hand surgeons were disinterested in learning the technique. They repeatedly said they preferred open fasciectomy and CCH was coming, which would be easier to learn and just as effective as PNF (American Society for Surgery of the Hand, Listserv, 2008). Then after CCH was released in February 2010, the interest for PNF suddenly increased. Cost appeared to be the major stumbling block for physicians who did not want to use CCH. That feeling was echoed in other parts of the world where health care systems are extremely cost conscious.

Having two different minimally invasive treatments available to treat DD enables a better shared decision-making process between the patient and their physicians. With PNF, it may be difficult or impossible to release very wide and thick cords, especially between the proximal finger crease and middle finger crease and in the first web space. Nerve injury rate is higher than CCH.

The technique for CCH is easier to learn and perform than PNF. Efficacy is high, complication rates are low (Gaston et al., 2015; Gilpin et al., 2010; Warwick et al., 2016) and satisfaction rates high (Bradley and Warwick, 2016; Kan et al., 2016; Warwick et al., 2015). With further evidence CCH became more efficient, allowing the treatment of up to two cords in a single treatment visit (as with PNF and surgery) and manipulation delayed for 24–72 hours after injection (Tursi et al., 2012; Coleman et al., 2014; Gaston et al., 2015; Pess, 2017).

The procedure can be performed in a clinic, freeing up the operating theatre for other patients. Surgical options are preserved. Physiotherapy is not needed after the procedure. The recurrence rate is >50% (Peimer et al., 2015; Werlinrud et al., 2018), and the procedure can be repeated (Arnold et al., 2020) or the patient may subsequently opt for LF. It is more expensive than PNF and the skin tear rate is higher (Denkler et al., 2017; Pess et al., 2012).

CCH is still available in selected locations worldwide through a Named Patient Special Access Program, although the cost will still restrict access for most patients and health systems. The proven utility of CCH for certain cords shows that ‘surgical drugs’ need to be developed and available worldwide at a cost that is affordable for all health care systems. A higher worldwide production rate should allow a further decrease in price, which in turn will make a novel surgical drug even more cost effective for the initial treatment, compared with LF, and a reasonable alternative for the treatment of recurrences*.*

## Are PNF and CCH equivalent?

One might intuitively feel that because the drug dissolved a segment of collagen (Figure 2) then the initial correction and eventual durability would be improved. However, randomized comparisons have shown that this hypothesis is not true (Table 1).

**Table 1.** PNF compared with needle fasciotomy in randomized studies.

Parameters	Abe et al. (2020)	Scherman et al. (2018)	Skov et al. (2017)	Strömberg et al. (2018)
Number of digits	70	50	50	152
Joints treated	PIP	MCP	PIP	MCP
Correction(CCH versus PNF)	60% versus 67%	89% versus 100%	69% versus 67%	90% versus 91%
Follow up (years)	3	3	3	2
Function	PNF > CCH	No difference	No difference	No difference

One must however interpret these studies with caution because the results of an randomised clinical trial (RCT) are only as generalizable as the inclusion criteria, exclusion criteria and dropouts. To have entered these trials, the patient would have to be suitable for *both* PNF *and* CCH (since larger cords or cords close to the PIP joint might have been deemed suitable by the recruiting surgeon’s personal Venn diagram (see below) to be suitable for CCH and not PNF. Nevertheless, one can at least conclude from these studies that for a cord that is suitable for both CCH and PNF, then one should choose PNF because it is cheaper, safer and has a quicker recovery. There are certain cords though – too dense for a surgeon to feel comfortable with PNF in a patient who wants to avoid surgery – where CCH would fulfil an unmet need.

## Needles or surgery: shared decision-making for patients and surgeons

Choosing the most suitable treatment is a shared process between the patient and the surgeon. Each patient has their own priorities and preferences, with their personal trade-offs for recurrence, complications, aesthetics, angular correction and rate of recovery (Kan et al., 2016). While the patient should have the final choice, that choice will inevitably be influenced by their surgeons’ preferences. Different surgeons will take a different perspective on the different treatment options that they present to a patient (McMillan et al., 2017). This is driven by training, experience, technical skill, attitude to risk, previous complications and finally the weight that the surgeon gives to a rapid rate of recovery and the later risk of recurrence (since these are mutually exclusive when comparing needles and surgery). So, a surgeon’s duty is to advise and consent a patient in a very individual way.

### Why is this patient seeking advice?

**Figure 3.**
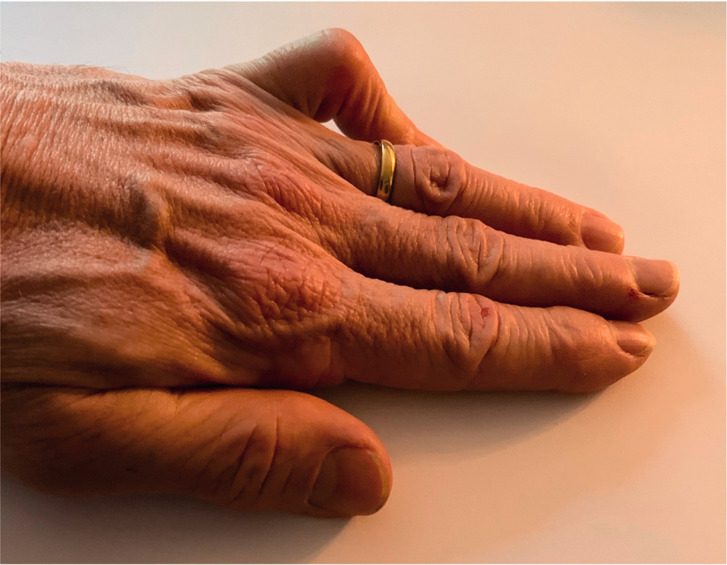
The ‘table top test’ is not necessarily a sensitive measure of disability or the need for surgery.

By no means all people with DD need or even want treatment. They may just want reassurance having seen relatives with a bigger problem (it is a genetic problem after all). There should be some identifiable problem against which to judge success. The ‘table top test’ (Figure 3) is not valid – one can get the finger flat on the table with a very marked deformity and yet have no functional problem. Treatment would subject the patient to all the inconvenience, expense and hazard of surgery for no benefit.

### Which treatment option matches the patient’s own disease?

Some cords are generally more suitable for PNF, such as a thin discrete cord or ‘twig’; whereas a thick cord with dense skin involvement or ‘log’ is not suitable for PNF but would be considered for a skin graft. A broader cord in a patient who does not want surgery and which for the surgeon’s skill level is too broad for a needle, might be more suitable, if available for CCH.

### Which treatment can this individual surgeon use confidently and competently in this particular patient?

Once the surgeon has determined what the patient really wants and what options are suitable, then the surgeon’s own Venn diagram must be added to the shared decision process (Figure 3).

**Figure 4.**
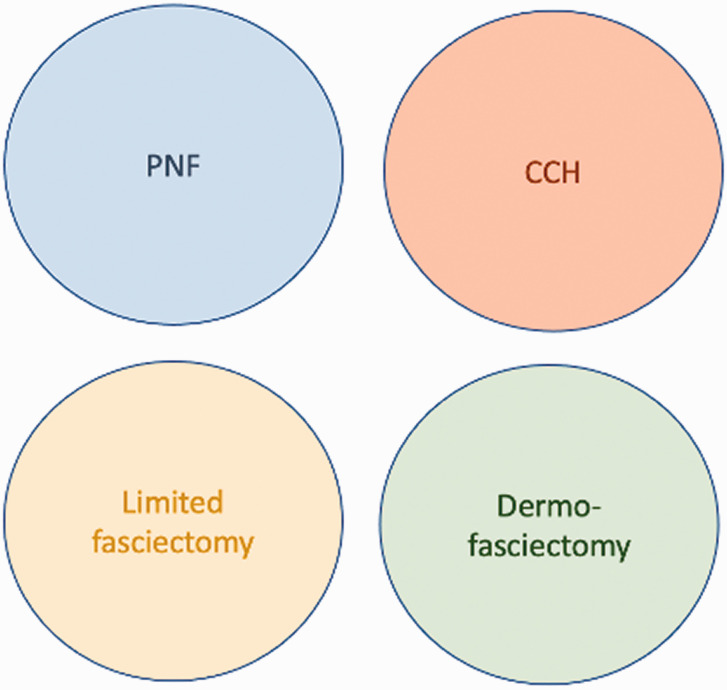
Venn diagram has infinite variations of overlap (cords that the surgeon considers are suitable for more than one treatment) and diameter (the proportion of each procedure performed).

If a surgeon is particularly skilled at PNF, which can take years especially for broader cords and cords near the PIP joint, and also feels that DF (i.e. skin grafting) is the best surgical procedure because of the lowest recurrence, then their personal Venn diagram may resemble Figure 4.

**Figure 5.**
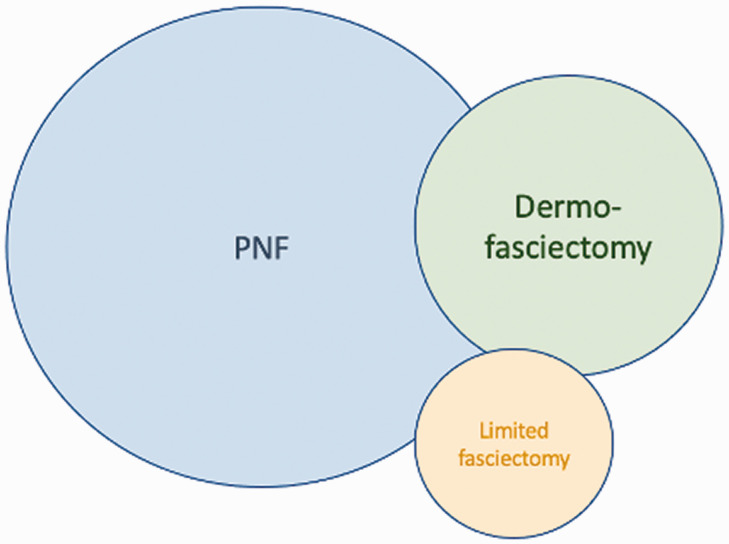
Venn diagram for the surgeon who prefers PNF and dermofasciectomy.

However, another surgeon may find PNF to be technically difficult, with an unsatisfying early correction and a disappointingly high recurrence, while also finding dermofasciectomy to be too invasive and technically demanding. Their personal Venn diagram would be different (Figure 5).

**Figure 6.**
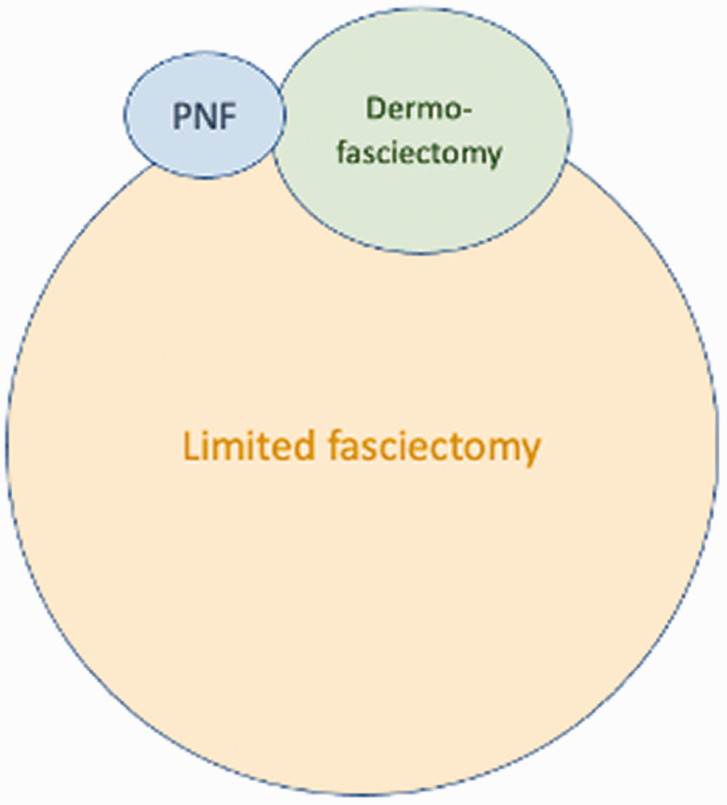
Venn diagram for the surgeon who prefers limited fasciectomy and is cautious about PNF and dermofasciectomy.

Recently, CCH was withdrawn from most healthcare systems. For those comfortable with its use and in a healthcare system able to afford it, CCH became a tool that uniquely allowed the treatment of cords that some surgeons found too large for PNF in patients who did not want to consider surgery (Figure 6).

**Figure 7.**
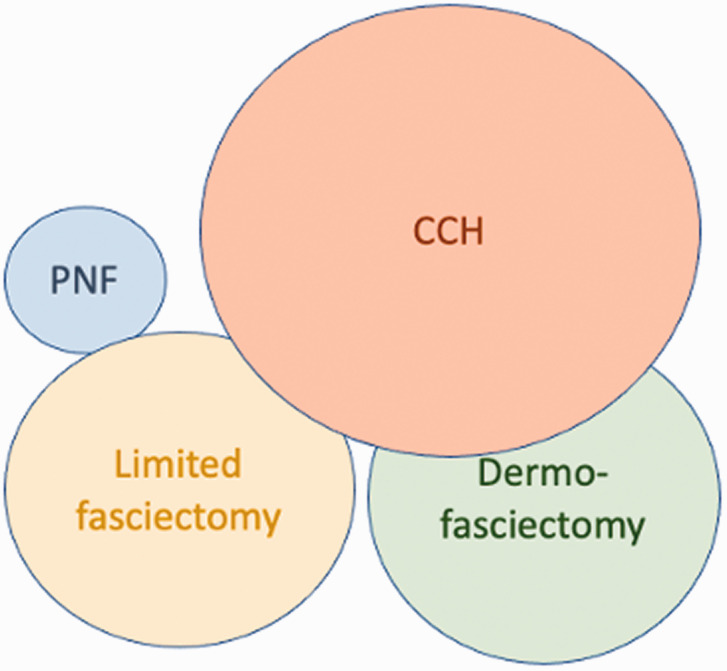
Venn diagram for a surgeon comfortable with CCH.

**Figure 8.**
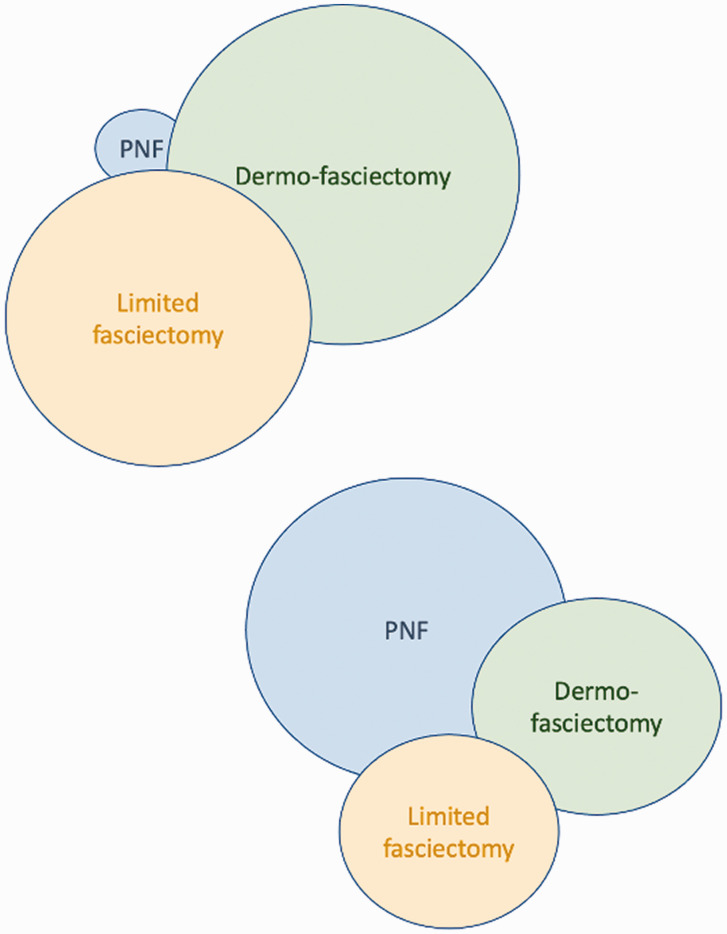
A figure shows the Venn diagram of a reluctant (or unskilled) user of PNF before the introduction of CCH (upper left), who then uses PNF far more frequently after the withdrawal of CCH- the second figure (lower right).

As the world emerges from the pandemic, there is a backlog of DD cases. Surgical resources have to be devoted to more important problems than painless bent fingers. Yet if the fingers are left too contracted for too long, especially at the PIP joint, the joints can become fixed. Given the short-term efficacy and safety of PNF, surgeons might reflect on their own Venn diagram and consider increasing their use of PNF as a means of protecting fingers from deterioration quickly, safely and cheaply. Those surgeons who have used CCH until it was withdrawn, may find that their favourable experience of using needles is in future reflected in more frequent use of PNF (Figures 7 and 8).

## Perspectives of Asia and Australia on PNF

### Asia

Until the early 1960s, people believed that DD was virtually confined to people of European descent. However, reports on DD from Asian and African countries have increased recently. An epidemiological study of DD in non-Caucasians conducted by Egawa (1980); for elderly people at a nursing home show a prevalence of 16%, although most were rated as Grade 0 according to Meyerding's criteria. Tajika et al. (2014) conducted a cross-sectional study of 401 subjects at a mountain village in the Gunma prefecture of Japan and found DD in 7% (12% in males and 3.8% in females). The prevalence showed a clear correlation with age: 0% in 40 s, 5.8% in 50 s, 6.3% in 60 s, 8.3% in 70 s and 11% in greater than 80 s. A similar study by Lanting et al. (2013) in the Netherlands reported a prevalence of 22% (26% in males and 19% in females), from which we might infer that the risk of DD in Japanese is lower than Caucasians. There appears to be a clear gender difference in disease severity in Japanese, with males accounting for over 95% of surgically treated cases in Japan. Involvement of three fingers or more is found in less than 20% of cases in Japan, suggesting less disease severity compared with Caucasian cases (Abe et al., 2004; Matsuzaki et al., 2010) assessed the influence of the ‘Dupuytren diathesis’ factors and found that bilateral hand involvement, little finger surgery, early onset, plantar fibrosis, knuckle pads and radial involvement significantly increase the risk of recurrence and/or extension in the Japanese population. This is consistent with findings in Caucasians (McFarlane, 1985).

There appears to be a generally lower risk for DD among other Asian countries. Lee et al. (2018) found in the Korean Health Insurance Review and Assessment service the mean annual prevalence of 42 per 100,000 in males and 23 per 100,000 in females, which is lower than Japanese, but still much higher compared with that in Taiwan (0.39–0.63 and 0.14–0.44 per 100,000 in males and females, respectively) (Jeh et al., 2015).

The majority of the cases in Japan had been treated with LF until 2015 according to Ministry of Health, Labour and Welfare's Diagnosis and Prognosis Combination database. The surgical outcomes were fairly good with 40% rated as very good, 31% good and the rest fair or poor using Tubiana’s criteria with 5 years or longer follow-up in most studies (Yoshida, 2004). This fairly good outcome might reflect much lower disease severity as compared with Caucasians cases. Regarding surgical complications, Uchida reported complex regional pain syndrome, nerve injury and skin necrosis as the most common events with risks of 11%, 7% and 7%, respectively (Uchida et al., 2002).

In 2015, the Japanese Pharmaceutical and Medical Devices Agency approved CCH. The treatment became popular because of simplicity and it quickly spread nationwide. In fact, more patients were treated by CCH injection rather than by surgery between 2015 and 2020 according to Ministry of Health, Labour and Welfare's Diagnosis and Prognosis Combination database. In order to compare hand function and direct medical cost between CCH and LF, we conducted a multicentre, non-randomized, controlled, observational study with two parallel groups (CeCORD-J study) (Hirata et al., 2017). Hirata et al. (2017) enrolled participants from all major referral centres in Japan with at least one hand surgery specialist. Patients with at least one PIP joint contracture and two or more fingers contracture in one hand participated. The primary outcome was the Hand10 score and EQ 5D-5 L score, patient satisfaction, degree of extension deficit and direct cost as secondary outcomes. Outcomes were evaluated up to 26 weeks after intervention. In this study, propensity score adjustment was used to balance difference in patient’s characteristics between the two groups. The CCH group scored a significantly better Hand10 at 2 weeks and higher EQ-5D-5 L at 8 weeks, but showed larger extension deficit of the PIP joint at 26 weeks. The surgical group had a higher cost. It is concluded that CCH injection provides better short-term hand function and cost effectiveness in Japan. Unfortunately, CCH imports were stopped in April 2020 and CCH is currently not available in Japan.

Given that cords in Japanese as compared with Caucasians are generally less severe, PNF appears to be a reasonable option to replace CCH. Abe conducted a retrospective study to compare treatment outcomes between CCH and PNF at 3 years and reported almost comparable results in both quick DASH score and recurrence rate (Abe, 2020). However, PNF has not yet gained popularity among Japanese hand surgeons and the vast majority of patients are now treated again by LF.

### Australia

The Australian, John Hueston, a plastic surgeon working at the Royal Melbourne Hospital made a great and early contribution to our thoughts on DD, based on his personal experience with over 6000 subjects (Hueston, 1963). PNF was introduced to Australia in 2006 with early positive reports but a slow uptake among surgeons. An analysis of 73 PNF and 52 limited LF found no significant differences in terms of immediate or medium-term deformity correction, tendon/nerve injury or circulatory complications and satisfaction scores. Postoperative infection rates were 7.6 times higher with LF when compared with PNF (Toppi et al., 2015). However, PNF has never been popular with Australian surgeons, perhaps because of the learning curve and the long-term surgical influence of John Hueston with his teaching of DF and ‘firebreaks’ on the surgical population. In a 2020 survey (Paynter et al., 2020), Australian surgeons believed that Tubiana’s treatment goal to correct the deformity was more important than shortening the post-treatment recovery. Less than half of the respondents performed PNF, while 70% performed CCH injections. Seventy-five per cent of hand surgeons felt that there was enough evidence to support the use of CCH for the treatment of DD. Almost 100% of surgeons performed LF, which was the most commonly performed treatment. When CCH was available, an Australian surgeons average Venn diagram was probably more consistent with Figure 6, whereas without CCH it would be more consistent with Figure 5.

In contrast to PNF, CCH became rapidly popular in Australia. The distribution of CCH in New Zealand was delayed because of administrative reasons and the update minimal. CCH was approved for use in Australia in 2013 and the drug went to market in 2014. The growth in the use (25% per year) peaked in 2020 at 2500 vials per year; in 2020 CCH represented 35% of the market share for the treatment of DD in the private health sector. The public healthcare sector within Australia was uniquely placed to introduce CCH because of the funding mechanisms (Medicare). CCH was delivered in 15 public hospitals within Australia, giving all patients with DD access to CCH irrespective of their ability to pay for the drug. CCH was confirmed to be a safe and effective option in Australian public health setting (Fletcher et al., 2019). CCH was also a much less expensive option than LF (Elliot et al., 2019; Sefton et al., 2018). In 2020 CCH was withdrawn from the Australasian market. Although a distributor (Tanner Pharmaceutical) has emerged, the cost per vial in Australia is AUD$10,989, which is prohibitive.

DD is one of the few benign conditions for which there is a potential role for radiotherapy (RT), with evidence to suggest that low dose palmar radiotherapy can reduce progression and the need for intervention. The DEPART (Dupuytren’s disease Evaluation of Preventive and Adjuvant Radiation Therapy) clinical trial in underway in Australia, which involves a randomization between intervention (PNF, CCH and LF) with radiotherapy and observation, with a preventative arm, and a post-intervention arm. Patients are randomized to either Observation or 30 Gy in 10 fractions of RT and followed for 5 years, with a primary efficacy endpoint of either definitive intervention or the development of a contracture at any joint in the affected hand of >20°. RT toxicity data as well as URAM and Quick-DASH patient-reported quality of life questionnaires are being collected.

## Summary

Worldwide, PNF is now used more frequently. CCH has been withdrawn from non-USA markets, which lessens its use. Different patients have different preferences, while different surgeons have different skills and opinions. The surgeon should fully consider the patient’s preference and should also, in view of the scarcity of surgical resource and the potential hazard of surgery, reconsider and expand the use of a needle rather than an operation. In the future, a cheaper, yet equally safe and effective alternative to CCH, will provide a useful clinical tool for those cords that, in the surgeon’s personal Venn diagram, are too challenging for PNF, but the patient prefers to avoid surgery.
